# Concurrent Topological Structure and Cross-Infill Angle Optimization for Material Extrusion Polymer Additive Manufacturing with Microstructure Modeling

**DOI:** 10.3390/mi13060852

**Published:** 2022-05-29

**Authors:** Ruixiao Tang, Chenghu Zhang, Jikai Liu

**Affiliations:** 1 Center for Advanced Jet Engineering Technologies (CaJET), Key Laboratory of High Efficiency and Clean Mechanical Manufacture (Ministry of Education), School of Mechanical Engineering, Shandong University, Jinan 250061, China; tangruixiao@mail.sdu.edu.cn (R.T.); zhangchenghu@mail.sdu.edu.cn (C.Z.); 2 Key National Demonstration Center for Experimental Mechanical Engineering Education, Shandong University, Jinan 250061, China

**Keywords:** homogenization, anisotropy, topology optimization, design for additive manufacturing

## Abstract

This paper contributes a concurrent topological structure and cross-infill angle optimization method for material extrusion type additive manufacturing (AM). This method features in modeling the process-induced material anisotropy through microscopic geometric modeling obtained by scanning electron micrographs. Numerical homogenization is performed to evaluate the equivalent effective properties of the 100-percentage cross-infilled local microstructures, and by introducing fitting functions, the relationship between equivalent effective material properties and varying cross-infill angles is empirically constructed. Then, optimization problems involving cross-infill angles as design variables are formulated, including concurrent optimization formulation. Numerical and experimental studies are conducted to illustrate the effectiveness of the proposed method. Both the numerical and experimental results demonstrate that the structural stiffness obtained by our proposed method has evidently improved.

## 1. Introduction

The past few decades have seen rapid development of additive manufacturing (AM) technology, including stereolithography apparatus (SLA), fused deposition modeling (FDM), powder bed fusion, etc. The layer-by-layer material deposition or solidification process eliminates the barriers in fabricating complex structures, and meanwhile, boosts the design activities that lead to a number of superior-performing mechanical structure examples [1,2,3]. On the other hand, this novel processing method has its limitations and challenges to design activities, such as material anisotropy [4,5], overhang free issue [6,7], residual stress [8,9], porous structures [10,11], multiscale design [12,13,14], etc. Among these topics, material anisotropy, as an inevitable AM feature, has been extensively studied for a long time.

The AM material anisotropy, mainly caused by the layer-based manufacturing process, is regarded as a process-induced feature [15]. Almost all AM processed materials, including metals, alloys, plastics, composites [16] and ceramics [17], exhibit certain levels of property anisotropy, while the widely adopted material extrusion-type AM process (such as FDM) for plastics and composites, present even more evident anisotropy than other AM processes [18]. Hence, the material extrusion process-related material anisotropy is the main focus of research. So far, material extrusion process-induced anisotropy has been widely discussed in the literature, and structural design methods addressing the anisotropic material properties that have been accordingly developed, as summarized below.

The build direction-related material anisotropy has been focused on for years. It has been experimentally found that processed plastics or composites commonly have lower moduli and tensile strength along the building direction. Optimization of the build direction for the improvement of mechanical performance was conducted in [19,20]. However, build direction optimization based on a fixed geometry only generates sub-optimal solutions. Tailoring the topological structure [21] while simultaneously optimizing the build direction is even more promising. Concurrent optimization considering density distribution and build direction for additively manufactured functionally graded lattice structures was investigated in [22]. In a recent study conducted by Li et al. [23], a concurrent structural topology optimization coupled with building direction for SLA-printed parts was presented. Additionally, the stress-constrained topology optimization for material extrusion-processed anisotropic materials was introduced in [24].

For material extraction-type AM processes, printing path optimization has emerged as an important topic due to its vital role in affecting the distribution of material anisotropy. As is widely recognized, the printed filaments or fiber-reinforced composites demonstrate stronger tensile modulus and strength along the printing path [25]. Early work in the field was aimed at formulating the printing path optimization based on the path orientations defined as discrete angle variables [26,27]. However, this strategy results in complex and disorganized local path directions, which un-trivialize the extraction of effective gap/overlap-free printing paths feasible for implementation. This issue can be relieved by applying smoothing filters [28]. To totally eliminate the path continuity concern, topology optimization methods with geometry-dependent contour-offset deposition path patterns were developed [4,5,29]. Recently, Sugiyama et al. [30] optimized the curved fiber trajectories based on preliminary stress field calculations. Liu et al. [31] presented an optimization method for gap/overlap-free design of carbon fiber-reinforced composites, wherein the fiber paths are represented by the equidistant iso-level set profiles. The above approaches performed topology optimization with printing path factors and showcased significant improvement of mechanical performance. However, the above works have focused on method development, while in-depth reasoning for layered path-induced varying microstructures and anisotropic material properties has not yet been addressed.

**Figure 1:**
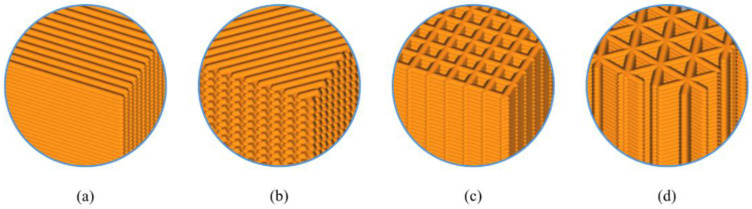
Illustration of different infill patterns with the highest infill density. From left to right, (**a**) lines, (**b**) cross, (**c**) cubic, and (**d**) triangles.

In the case of using commercial 3D printing pre-processing, the 3D-printing deposition path is restricted to the selectable infill patterns, e.g., one or two contour-offsets plus the infill patterns of lines, cross, cubic, or triangles (see Figure 1), and therefore, the specific infill patterns and the microscopic interactions amongst the filaments are determinant of the equivalent macroscopic material anisotropy [32]. Aloyaydi et al. [33] presented an investigation on the effect of infill patterns on the mechanical response of 3D-printed specimens, wherein the grid-infill/cross-infill pattern had shown the highest tensile strength due to the special interlacement between layers. Similar studies were reported by [34,35]. Though widely studied (especially when including experiments), it seems that the way infill patterns influence the mechanical properties of AM parts has not yet been fully revealed. The in-depth reasoning for the interactions between layered filaments deserves careful attention, and the linkage between the microstructures and macroscopic equivalent mechanical properties should be calibrated. The above knowledge plays a key role in realizing concurrent optimization on the structural topology and the infill pattern parameters.

To address the above issues, this paper presents an optimization method for material extrusion polymer AM with cross infills, which is involved in quantifying the interactions between filaments and then linking the microscopic configurations to the macro-equivalent anisotropic properties. Specifically, the periodic microstructures subject to different cross-infill angles are constructed facilitated by experimental calibrations. Numerical homogenization is then implemented to evaluate material effective properties, and mechanical tests on the FDM-processed specimens with different cross-infill angles are conducted to validate the homogenization results. Finally, two types of optimization problems are studied for optimization of the cross-infill angles and the structural topology.

The remainder of this paper is organized as follows. Section 2 illustrates the method details of the proposed optimization method for material extrusion polymer AM. Meanwhile, the optimization problems are formulated, and the related sensitivities are derived. Section 3 demonstrated two case studies combined with experimental validation to show the effectiveness of the proposed method. Finally, Section 4 gives the conclusions for this study.

## 2. Details of the Proposed Method

### 2.1. Geometric Modeling and Numerical Homogenization

**Figure 2:**
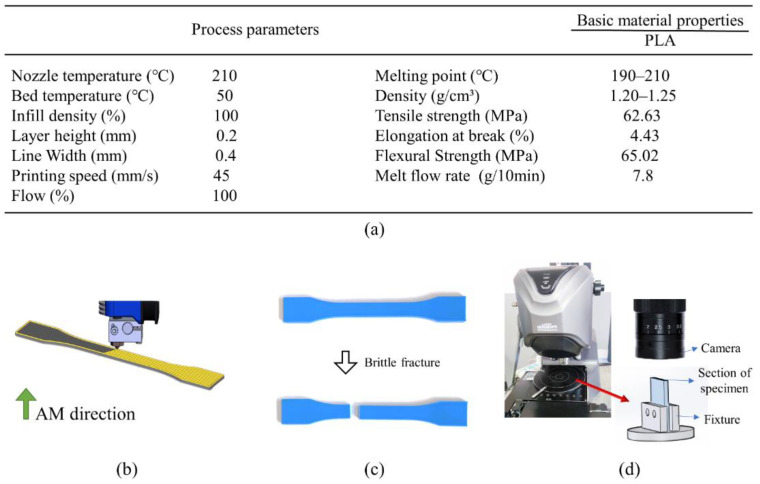
Preparation of the specimens: (**a**) printing process parameters and material properties, (**b**) printing setup of the specimens, (**c**) the additive manufacturing specimens, and (**d**) laser scanning microscopy.

**Figure 3:**
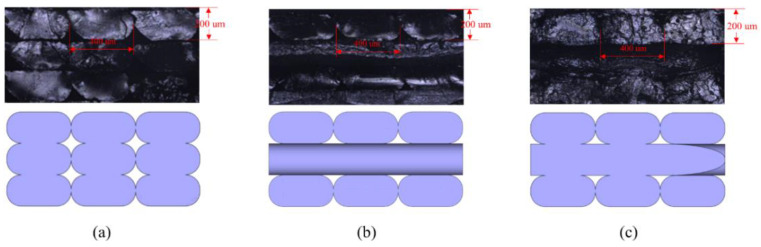
The scanning electron micrographs of the fracture surfaces and the constructed geometric models with three typical cross-infill angles: (**a**) 0 degrees, (**b**) 90 degrees, and (**c**) an in-between cross-infill.

**Figure 4:**
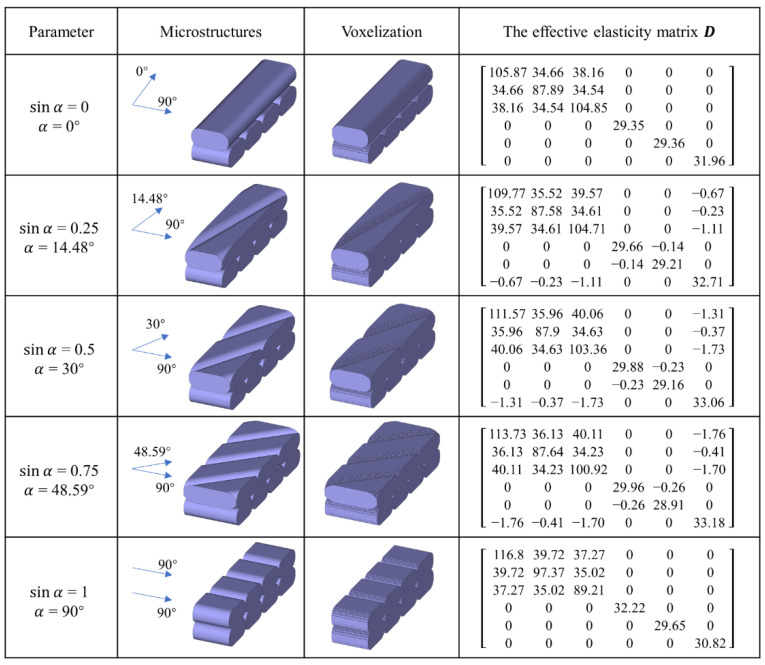
The representative periodic microstructures and their effective elasticity properties.

Layer-by-layer processing in additive manufacturing leads to the evident anisotropy property of the printed materials and it is worthwhile to reason the material anisotropy by fine-tuning the microstructures through differently oriented cross-infill path patterns. To start with, several specimens are prepared by material extrusion polymer additive manufacturing with different cross-infill angles and an infill density of 100%. The material used is 1.75 mm PLA filaments from JGAURORA, China. The printing-process parameters and material properties are illustrated in Figure 2a, and the printing setup is shown in Figure 2b. Next, the printed samples were stored in liquid nitrogen at low temperature for hours and broken with brittle fracture to obtain flat breakage surfaces, as shown in Figure 2c. Afterwards, laser scanning microscopy (VK-X200K, Keyence, Japan) is utilized to get the microscopic configurations of the printed cross-infill materials at 200 times magnification. At least three fracture surfaces were developed for each cross-infill angle. Figure 3 summarizes the scanning electron micrographs of specimens with three typical cross-infill angles, namely 0 degrees in Figure 3a, 90 degrees in Figure 3b, and an in-between cross-infill angle as shown in Figure 3c. It can be seen that the filaments align well along the printing path, but voids exist between adjacent filaments due to the elliptic cross-section shape, which results in the macroscopic anisotropy mechanical properties. According to the microscopic images and the measured dimensional ratios, the geometric models can be established. Next, numerical homogenization [36] is applied in order to obtain effective elasticity properties by approximating the periodic microstructures as homogeneous materials. It is noted that the key to numerical homogenization is to ensure the periodicity of microstructures. Thus, we calibrate the representative unit cell size according to the cross-infill angles in order to satisfy the periodic boundary conditions. On the premise of generality, in this study, we define the cross-infill angle 90°–α as the filament in lower layer is fixed to 90 degrees, the filament in the upper layer is α, and the 90°–α layout repeats for other layers. Figure 4 shows the definition of cross-infill angles and the microstructure geometric models. The homogenized effective elastic matrices of the microstructures are also provided. It is noted that a virtual base material property of $E_{0}$ = 100 and μ = 0.3 is assigned.

In order to carry out cross-infill angle optimization, it is necessary to parameterize the material properties, i.e., to build the mathematical relationship between the elastic properties of the cross-infill materials and cross-infill angles. A polynomial interpolation function is employed to describe the relationship between the homogenized effective elastic matrix D and the sine value of α, as:

(1) $$D_{ij}=a_{1}+a_{2}\left(sin\;\alpha \right)+a_{3}{\left(sin\;\alpha \right)}^{2}+a_{4}{\left(sin\;\alpha \right)}^{3}+a_{5}{\left(sin\;\alpha \right)}^{4},$$

where $D_{ij}$ is the constants of the homogenized effective elastic matrix. $a_{i}$ (*i* = 1~5) denotes the fitting coefficients obtained by the least squares-based regression analysis from the above 5 samples (from Figure 4). The fitting coefficients for the elastic constants are given in Table 1.

### 2.2. Experimental Validation

The last subsection constructs the geometric models for the periodic microstructures of the cross-infill materials and the equivalent elastic properties are obtained via the homogenization method. To verify the effectiveness of the established models, numerical and experimental tests were carried out on the MBB beam.

**Figure 5:**
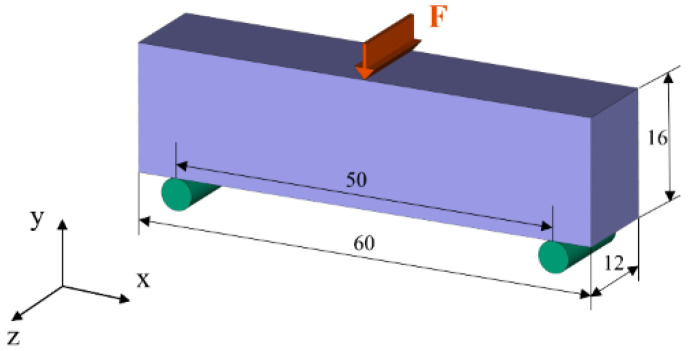
The boundary conditions of the MBB beam.

**Figure 6:**
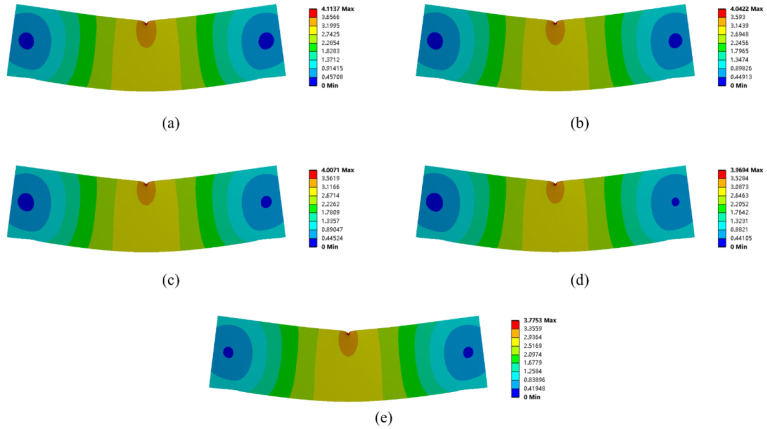
The deformation nephograms of the MBB beam with the assigned effective elastic properties of cross-infill materials. The cross-infill angles are (**a**) 90°–0°, (**b**) 90°–14.48°, (**c**) 90°–30°, (**d**) 90°–48.59°, and (**e**) 90°–90°, respectively.

Figure 5 depicts the boundary conditions of the MBB beam. The beam is supported at two foot corners, and a distributed force is applied to the middle of the beam. With the assigned homogenized elastic properties (from Figure 4), the deformation nephograms of the MBB beam are simulated in ANSYS through static structure analysis, as shown in Figure 6. Specially, the total magnitude of the distributed forces is set as 10.

**Figure 7:**
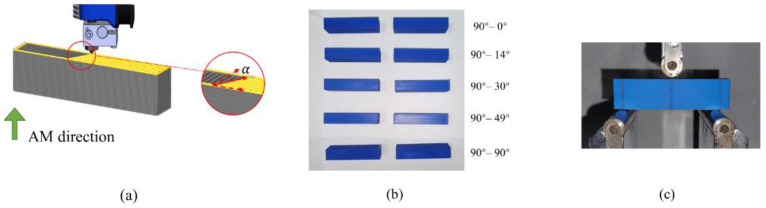
Mechanical test details: (**a**) the sample printing setup, (**b**) the fabricated experiment specimens, and (**c**) the test setup.

The details of the experimental test are as follows. Figure 7a demonstrates the AM direction of the experiment samples. Figure 7b shows the fabricated experiment specimens from the top view. The overall structure size of each specimen is 60 mm × 16 mm × 12 mm. Each type of experimental specimen was printed twice for mechanical tests to prevent contingency of the experiments. The experiments were performed with a universal mechanical testing machine with a loading speed of 2 mm/min. The testing setup is shown in Figure 7c.

**Figure 8:**
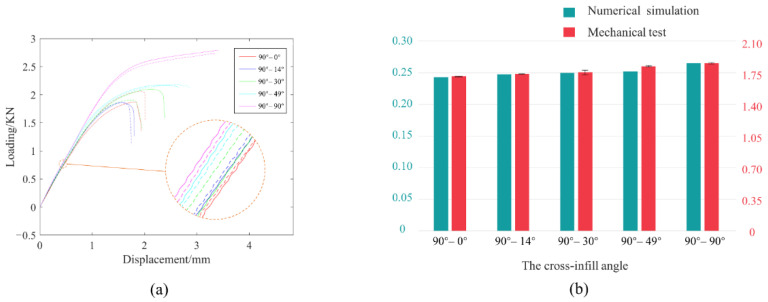
Mechanical test results and their comparison with numerical simulations. (**a**) The force-displacement curves of the mechanical tests. (**b**) The comparison between numerical simulation and mechanical test results.

Figure 8a shows the force-displacement curves for all the tests. Then, we evaluate the linear segments of the experimental curves to extract the load-displacement ratios, and the results are compared with the ratios of the applied force to displacement from the numerical simulations (in Figure 6). Figure 8b summarizes the comparison results, wherein satisfactory consistency can be reached. It is noteworthy that the base material properties in the numerical simulations are virtual, while altering the applied Young’s modulus only scales the *y*-axis coordinates while not affecting the layout of the inclination ratio bars. Therefore, not applying the true fundamental material properties when performing representative unit homogenization and structural finite element analysis does not harm the truthfulness of the comparison, and consistency between the numerical predictions and the experimental tests can be reached.

### 2.3. Formulation and Sensitivity Analysis of the Optimization Problem

Based on the proposed fitting model that maps the cross-infill angle to the material effective properties, two types of structural optimization problems are considered and formulated, namely, the angle optimization considering cross-infill and the concurrent optimization considering cross-infill angles and structural topology.

#### 2.3.1. Angle Optimization Considering Cross-Infill

**Figure 9:**
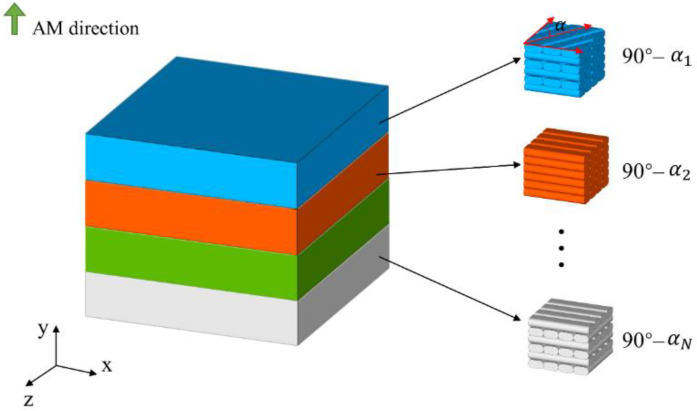
A 3D structure is divided into N regions with different cross-infill angles.

In the first optimization problem, we consider a minimum compliance optimization problem for 3D structures with a cross-infill path pattern. As shown in Figure 9, a 3D structure is divided into N regions along the AM direction, and the cross-infill angle 90°-$\alpha_{i}$ in each region is the design variable to be optimized. The mathematical formulation of the topology optimization problem is stated as below:

(2) $$\mathrm{find}:\;\xi ={\left[\alpha_{1},\alpha_{2},\ldots ,\alpha_{N}\right]}^{T}.\;\min:\;C=U^{T}KU=\overset{n}{\underset{e=1}{\sum }}U_{e}^{T}K_{e}U_{e}.\;s.t.:\;F=KU\;,\;0{}^{\circ}\leq \alpha_{i}\;\leq \;90{}^{\circ},\;\;i=1,2,\ldots ,N.\;$$

where ξ is the design variable vector that collects the design variable in each region; C denotes the structural compliance; K and ***U*** are the global stiffness matrix and nodal displacement; n is the total number of elements; $K_{e}$ and $U_{e}$ are the elemental stiffness matrix and nodal displacement vector, respectively; and F is the global force vector.

According to the adjoint method, the derivatives of the objective function with respect to the design variable $\alpha_{i}$ is calculated as:

(3) $$\frac{\partial C}{\partial \alpha_{i}}=-U^{T}\frac{\partial K}{\partial \alpha_{i}}U.$$

Considering that $\alpha_{i}$ only has effects on the stiffness matrix K in the i-th region, Equation (3) could be simplified as:

(4) $$\frac{\partial C}{\partial \alpha_{i}}=-\underset{e\in R_{i}}{\sum }U_{e}^{T}\frac{\partial K_{e}}{\partial \alpha_{i}}U_{e}=-\underset{e\in R_{i}}{\sum }U_{e}^{T}\left(\int_{\Omega_{e}}B^{T}\frac{\partial D}{\partial \alpha_{i}}Bd\Omega_{e}\right)U_{e},$$

where $R_{i}$ is the set of elements for which the element belongs to the i-th region, B is the strain–displacement matrix, and $\Omega_{e}$ is the elemental domain. The effective elastic matrix D and the derivative $\frac{\partial D}{\partial \alpha_{i}}$ are obtained by assembling the interpolation functions of Equation (1).

Based on the sensitivity information, this optimization problem can be solved by means of the method of moving asymptotes (MMA) [37].

#### 2.3.2. Concurrent Optimization for Cross-Infill Angles and Structural Topology

In the second optimization problem, a concurrent 3D optimization problem considering cross-infill angles and structural topology is studied. Thus, the design variable vector χ that collects the density value $x_{e}$ of each element and the cross-infill angle α of the overall structure is written as:

(5) $$\chi ={\left[x_{1},x_{2},\ldots ,x_{n},\alpha \right]}^{T}.$$

When the element density is assumed to be $x_{e}$, the elemental stiffness matrix $K_{e}$ can be interpolated using the modified Solid Isotropic Material with Penalization (SIMP) model [38] and calculated as:

(6) $$K_{e}=\left(E_{min}+{\left(x_{e}\right)}^{p}(1-E_{min}\right))K_{0},$$

where $K_{0}$ is the stiffness matrix of a full solid element, $E_{min}$ is a very small value to prevent the stiffness matrix from becoming singular, and p is the penalization factor to promote convergence to a 0–1 solution—in this study we use p=3.

Further, the topology optimization problem is formulated as:

(7) $$\mathrm{find}:\;\chi ={\left[x_{1},x_{2},\ldots ,x_{n},\alpha \right]}^{T}.\;\min:\;C=U^{T}KU=\overset{n}{\underset{e=1}{\sum }}U_{e}^{T}K_{e}U_{e}.\;s.t.:\;F=KU,\;\frac{V}{V_{0}}=\frac{1}{n}\overset{n}{\underset{e=1}{\sum }}x_{e}\;\leq \;f,\;0\leq x_{e}\leq \;1,\;\;e=1,2,\ldots ,n.\;0{}^{\circ}\leq \alpha \leq \;90{}^{\circ}.$$

where χ is the design variable vector that collects the density value $x_{e}$ of each element and the cross-infill angle α of the overall structure; V and $V_{0}$ are the structure volume and design domain volume; and f is the prescribed volume fraction limit.

Similarly, when the gradient-based algorithm is used to solve the optimization problem, the sensitivity information of the objective and constraint functions with respect to the design variables is necessary.

The derivatives of the objective function C with respect to the element density value $x_{e}$ are calculated as:

(8) $$\frac{\partial C}{\partial x_{e}}=-U_{e}^{T}\frac{\partial K_{e}}{\partial x_{e}}U_{e}.$$

Combining with (6), Equation (8) is expressed as:

(9) $$\frac{\partial C}{\partial x_{e}}=-p{\left(x_{e}\right)}^{p-1}(1-E_{min})\;U_{e}^{T}K_{0}U_{e}=-p{\left(x_{e}\right)}^{p-1}(1-E_{min})\;U_{e}^{T}\left(\int_{\Omega_{e}}B^{T}DBd\Omega_{e}\;\right)U_{e}.\;$$

The derivatives of the objective function C with respect to the cross-infill angle α is calculated as:

(10) $$\frac{\partial C}{\partial \alpha }=-U^{T}\frac{\partial K}{\partial \alpha }U=-\overset{n}{\underset{e=1}{\sum }}U_{e}^{T}\frac{\partial K_{e}}{\partial \alpha }U_{e}.$$

Combining with (6), Equation (10) is expressed as:

(11) $$\frac{\partial C}{\partial \alpha }=-\overset{n}{\underset{e=1}{\sum }}\left(E_{min}+{\left(x_{e}\right)}^{p}(1-E_{min}\right)\;)U_{e}^{T}\left(\int_{\Omega_{e}}B^{T}\frac{\partial D}{\partial \alpha }Bd\Omega_{e}\;\right)U_{e}.$$

The sensitivity of the structural volume V with respect to the element density value $x_{e}$ and cross-infill angle α can be calculated as:

(12) $$\frac{\partial V}{\partial x_{e}}=1,$$

(13) $$\frac{\partial V}{\partial \alpha }=0.$$

## 3. Case Studies

### 3.1. Example 1

**Figure 10:**
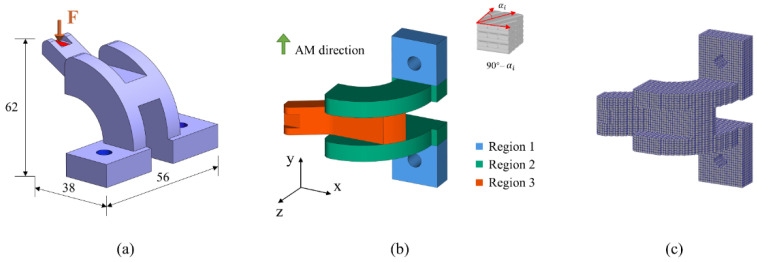
Illustration of the first example. (**a**) The dimension and boundary conditions of the bracket; (**b**) the AM direction and region divisions; (**c**) voxelization of the bracket.

In the first numerical example, a 3D bracket is studied. The dimension and boundary conditions of the bracket are shown in Figure 10a. The holes marked in blue are fixed, and a distributing force F = 20 is applied vertically to the top area marked in red. The AM direction and region divisions are illustrated in Figure 10b. The optimization is to design the cross-infill angle in each region for minimum structural compliance. Then, the design domain is voxelated and divided into 52 × 38 × 62 grids as shown in Figure 10c. The optimization begins with an initial guess of the three regions all being filled with 90°–90° cross-infill paths. The optimization process terminates when the optimization loop is larger than 20.

**Figure 11:**
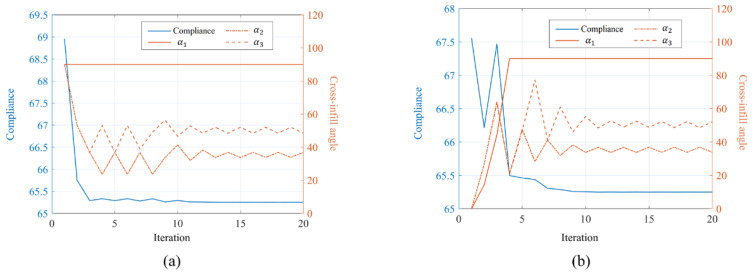
The convergence histories of the optimization processes from cross-infill initial guesses of (**a**) 90°–90° and (**b**) 90°–0°.

**Figure 12:**
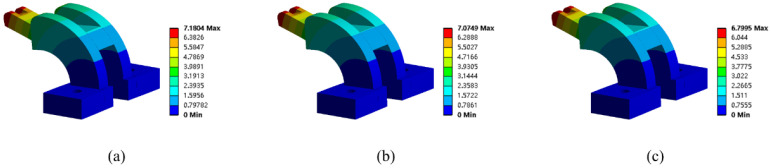
The deformation nephograms of the structures with different cross-infills: (**a**) 90°–90°, (**b**) 90°–0°, and (**c**) optimized cross-infill angles.

The convergence history of the optimization process is depicted in Figure 11a. The compliance curve eventually converges to 90°–90°, 90°–37°, and 90°–49° distribution in the three divided resigns, respectively. The optimization results demonstrated a 5% reduction in structural compliance compared to the initial guess. Meanwhile, to evaluate the effect of different initial guesses on the optimization result, the optimization is also performed with an initial guess of the three regions being filled with 90°–0° cross-infill paths. The convergence history is shown in Figure 11b. It is found that the same optimization results are found from the new initial guess. Meanwhile, the deformation nephograms of the structures are shown in Figure 12, wherein the structure is filled with uniform cross-infill angles of (a) 90°–90°, (b) 90°–0°, and (c) optimized cross-infill angles, respectively.

### 3.2. Example 2

**Figure 13:**
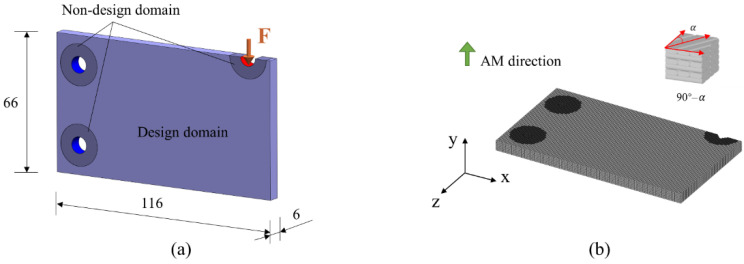
Illustration of the second example. (**a**) The dimension and boundary conditions of the cantilever; (**b**) the AM direction and voxelization.

In the second numerical example, the concurrent optimization for a cantilever case considering cross-infill angle and structural topology is studied. The dimension and boundary conditions of the cantilever are shown in Figure 13a. The left holes of the structure are fixed and a distribution load F = 10 is applied to the top-right hole area. Figure 13b illustrates the AM direction and the voxelization by 116 × 66 × 6 grids. The total volume constraint is f=0.5. The optimization begins with an initial guess of the structure being filled with a 90°–90° cross-infill, and the density value of each element is assigned to f. In order to prevent the occurrence of checkerboard pattern and ensure the size control for additive manufacturing, the well-known density filter [39] is adopted with a radius of four times the element size. The maximum iteration number is 100.

**Figure 14:**
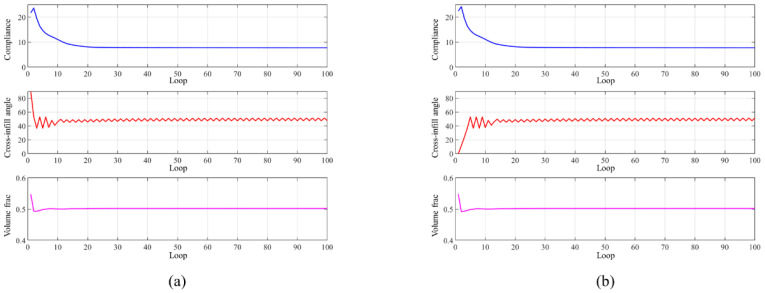
The convergence histories of the optimization processes from cross-infill initial guesses of (**a**) 90°–90° and (**b**) 90°–0°.

The convergence history of the optimization process is shown in Figure 14a. The compliance iteration curve eventually converges to 7.71, and the optimized angle α = 47.4° or 51.44° is obtained. The topology optimization performed with the initial guess of a 90°–0° cross-infill angle combination was also studied, providing the same optimization result as shown in Figure 14b.

**Figure 15:**
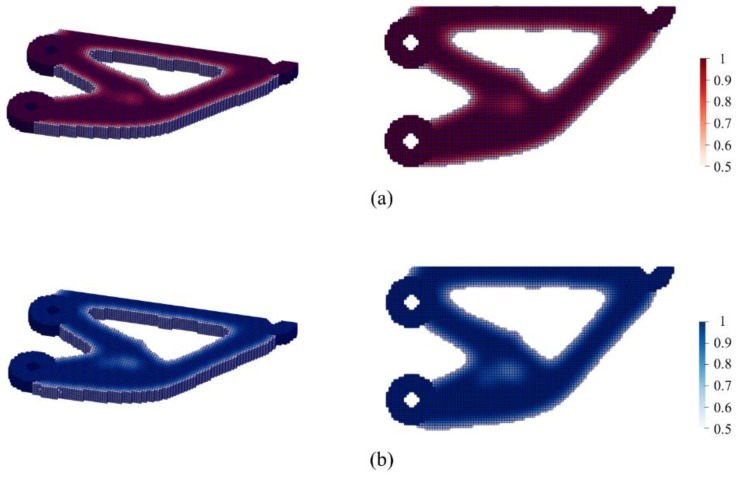
Comparison of the optimized cantilevers by using (**a**) our method and (**b**) the SIMP method.

The optimized cantilever is shown in Figure 15a. For the purpose of comparison, the optimization results obtained by the SIMP method are illustrated in Figure 15b without consideration of any infill patterns (using an isotropic material model). To obtain comparable structural compliance values, the SIMP-based design was assigned the cross-infill path pattern with crossing angles of 90°–0° and 90°–90°, resulting in structural compliances of 8.10 and 7.97, respectively.

**Figure 16:**
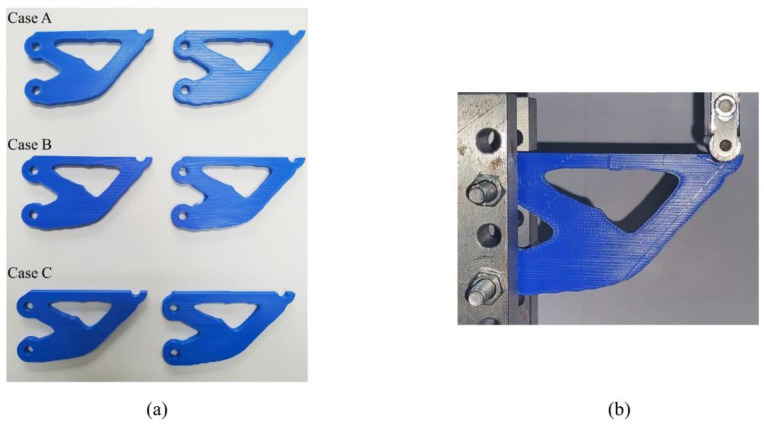
3D printing and mechanical test results: (**a**) the 3D printed experimental specimens, and (**b**) the mechanical test setup.

To further validate the effectiveness of the proposed method, additive manufacturing and mechanical tests were performed on the above three numerical results. Figure 16a shows the 3D printed experimental specimens. The structures in Case A were obtained by using our method and fabricated with the optimized cross-infill angles of 90°–47°. The structures in Case B and Case C were obtained using the SIMP method, and fabricated with 90°–0° and 90°–90° cross-infill angles. The overall structure size of each specimen is 90 mm × 48 mm × 5.5 mm. The creation of each type of experimental specimen was repeated twice to prevent experimental contingency.

**Figure 17:**
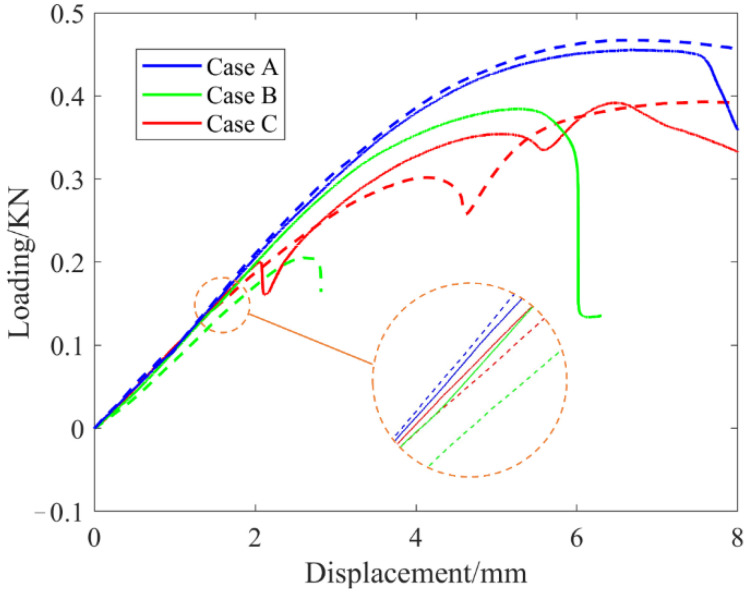
The load-displacement curves from the mechanical tests.

The experiments were performed with the WDM-100 electronic universal testing machine with a loading speed of 2 mm/min. The setup of the bending experiments for the cantilever beams is shown in Figure 16b. The load-displacement curves are illustrated in Figure 17. It is found that the cantilever optimized by our method is stiffer than the others, which confirmed the numerical predictions.

## 4. Conclusions

This paper evaluates the microstructures of cross-infilled 3D printing parts through scanning electron micrographs and builds the linkage between the material effective properties and the microstructures through numerical homogenization and regression analysis. Hence, the reasoning on process-induced material anisotropy is performed and the gained knowledge plays avital role in supporting structural optimization with cross-infill angle variables. Numerical results and mechanical tests illustrate the effectiveness of our proposed method in gaining improved structural stiffness.

On the other hand, the assumptions about the isotropy and tension-compression homogeneity of the AM raw materials are not sufficiently rigorous. Enhancing the accuracy of the constitutive modeling will be focused on in our forthcoming work.

## Figures and Tables

**Table 1:** The fitting coefficients for the elastic constants.

	$a_{1}$	$a_{2}$	$a_{3}$	$a_{4}$	$a_{5}$
$D_{11}$	105.90	24.99	−50.49	56.80	−20.37
$D_{12}$	34.66	0.94	21.40	−55.04	37.76
$D_{13}$	38.16	11.05	−32.69	52.00	−31.25
$D_{16}$	0.00	−4.39	12.60	−28.05	19.84
$D_{22}$	87.89	−15.89	101.10	−20.14	12.57
$D_{23}$	34.54	−2.09	17.08	−35.63	21.12
$D_{26}$	0.00	−1.43	3.13	−5.33	3.63
$D_{33}$	104.80	10.32	−71.51	134.10	−88.53
$D_{36}$	0.00	−6.07	8.95	−12.05	9.17
$D_{44}$	29.35	−1.02	17.06	−38.45	25.28
$D_{45}$	0.00	−0.87	1.93	−3.41	2.35
$D_{55}$	29.36	−2.69	14.13	−27.04	15.89
$D_{66}$	31.96	6.45	−22.31	40.53	−25.81
